# Primary squamous cell carcinoma of lung in a 13-year-old boy: a case report

**DOI:** 10.1186/1757-1626-1-123

**Published:** 2008-08-22

**Authors:** Jeff F Wang, Bo wang, Joshua A Jansen, Eric E Santos, Deba P Sarma

**Affiliations:** 1 Department of Pathology, Creighton University Medical Center, Omaha, Nebraska, USA; 2 St. Margaret's Hospital, Spring Valley, Illinois, USA

## Abstract

We are reporting a very rare case of primary bronchogenic squamous cell carcinoma (SCC) with bone metastasis in a 13-year-old boy. A brief review of the English literature on this rare neoplasm in childhood is presented.

## Case presentation

**Figure 1:**
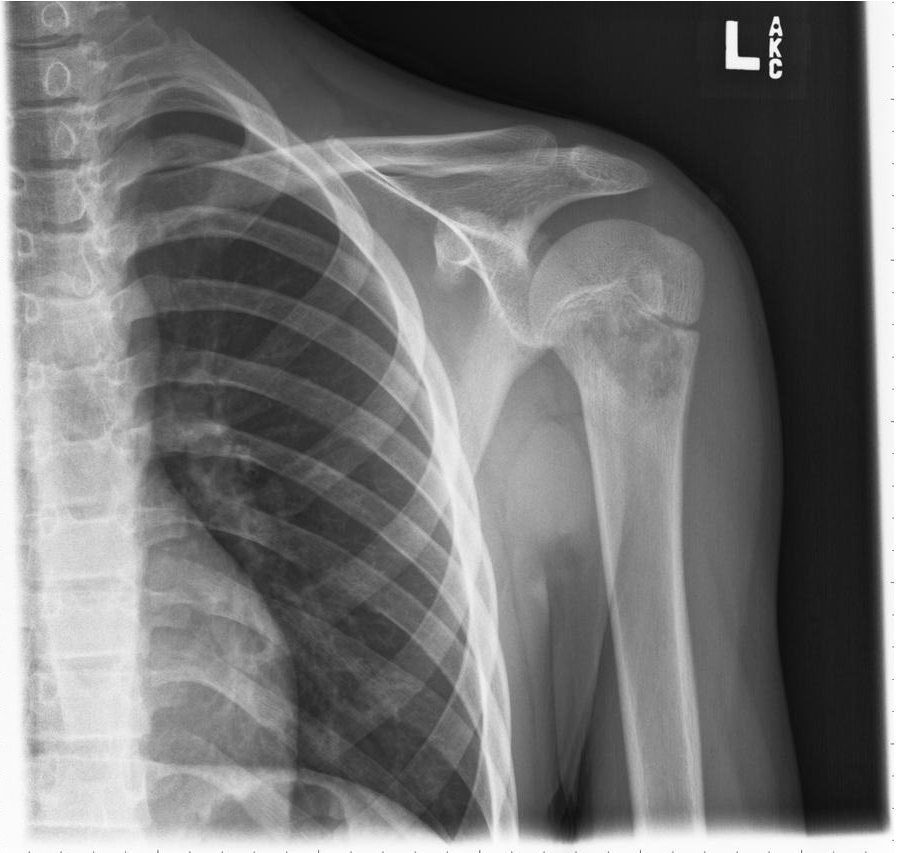
X-ray of left shoulder shows destructive lesion of humerus.

**Figure 2:**
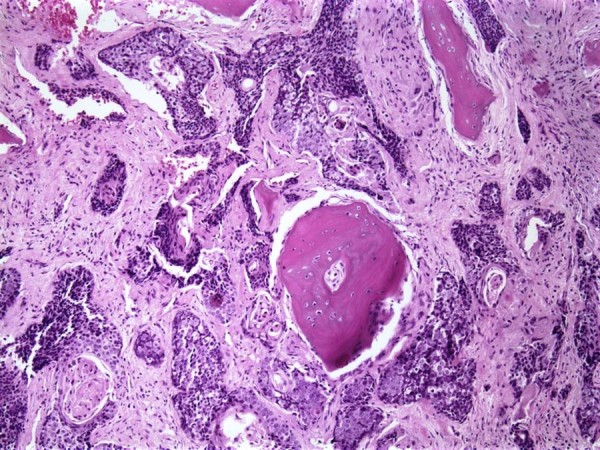
Invasive islands and nests of squamous cells with basaloid appearance (Hematoxylin and eosin stain, 10×).

**Figure 3:**
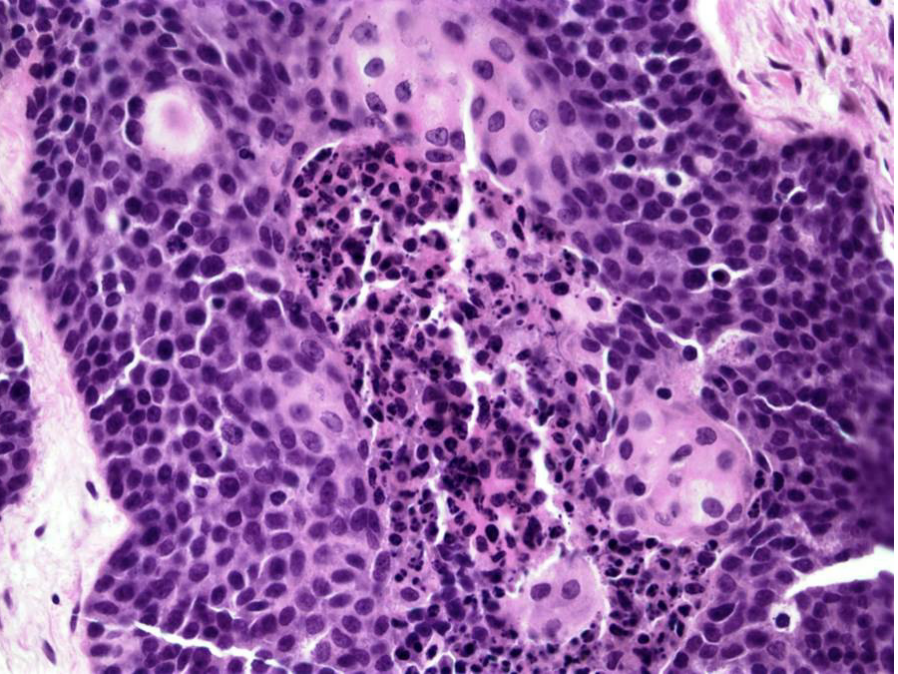
Squamous cells with prominent nucleoli, numerous mitoses and necrosis (Hematoxylin and eosin stain, 20×).

A 13-year-old boy presented with a two-month history of left shoulder pain. Radiographs followed by MRI demonstrated a destructive lesion of the metaphysis of the proximal third of the left humerus [Fig 1]. The metaphysis of the humerus was replaced by a 5-cm tumor. There was some erosion of the cortex with minimal soft tissue extension by the tumor. The initial impression of the lesion was an osteosarcoma, however, an open biopsy revealed metastatic squamous cell carcinoma. The tumor showed islands and nests of squamous cells with a basaloid appearance at the periphery with maturation and squamous pearl formation in the center [Fig 2]. The cells appeared anaplastic with prominent nucleoli, numerous mitoses and focal necrosis [Fig 3].

**Figure 4:**
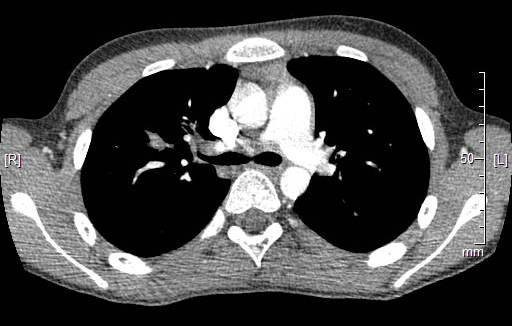
Chest CT confirms the neoplasm in the right lung.

Molecular cytogenetic studies on the tumor were positive for rearrangement of the NUT1 region in 54% of the interphase cells. Additional interphase FISH studies were negative for the NUT/BDR4 fusion. The patient underwent a PET-CT that showed multiple foci of increased uptake in the right upper lobe of the lung. Additional abnormal uptake was also noted at T1, T8, T10, and T11, left proximal humerus, right hilar nodes and right acetabulum. A subsequent chest CT confirmed. the malignant lung neoplasm [Fig. 4] Since lung cancer is an extremely rare childhood neoplasm, radon tests are performed in the family home and the results were negative. Upon further questioning, a history of lung cancer in both grandmothers was uncovered. One grandfather also had a history of non-Hodgkin lymphoma and pancreatic cancer. The patient was treated with various chemotherapeutic agents. There was no significant clinical improvement and he died one year later.

## Discussion

Primary lung cancer is the most frequently diagnosed cancer in the USA and the most common cause of cancer mortality worldwide. It occurs most often between the ages of 40 and 70 years, with a peak incidence in the fifties and sixties. Only 2% of all cases appear before the age of 40. Primary lung cancer in childhood is a rare entity and primary bronchogenic squamous cell carcinoma is extremely rare. To our knowledge, only eight cases of primary bronchogenic squamous cell carcinoma in childhood have been reported in English literature.

Pulmonary squamous cell carcinoma is most commonly found in men and is closely correlated with a smoking history. Histologically, the tumor is characterized by the presence of keratinization and intercellular bridges. It is graded according to the degree of keratinization, squamous pearl formation, or intercellular bridges. These features are obvious in the well-differentiated tumors but only focally demonstrated in the poorly-differentiated tumors.

Cayler et al [1] in 1951 reported 16 cases of primary carcinoma of the lung in children less than 15 years of age Primary bronchogenic squamous cell carcinoma is extremely rare in childhood and adolescence. Eight histologically confirmed cases reported in the English literature [2-9] are summarized along with the present case [Table 1].

In 1974, Niitu et al [2] reported one case of squamous cell carcinoma in a boy and reviewed the world literature and found 39 cases of primary lung cancer in children less than 16 years of age. These cases included two cases of bronchogenic squamous cell carcinoma [6,8]. Since then only five additional cases of primary brochogenic SCC have been reported, including one case with substantial family history of cancer [[3-5,7], and [9]]. Most of the patients (eight out of nine) are boys. The clinical presentation of these bronchial cancers varies with the extent of the primary tumor. In our case, the patient presented with bone pain due to metastasis. Four of the reported cases presented with recurrent pneumopathies and hemoptysis. Three of the reported cases were incidentally found by chest x-ray. One case was discovered by routine chest radiograph. There have been no clearly identified risk factors. There is no standard treatment and management essentially depends on the initial findings of the extent of tumor and the presence of metastases. Generally, the prognosis is poor due to metastatic disease.

The high frequency of p53 mutations have been seen in all histological types of lung carcinoma. Loss of tumor suppressor gene RB, inactivation of CDK-inhibitor, and overexpression of epidermal growth-factor receptor might contribute to the development of neoplasm. There are no p53 mutations or loss of tumor suppressor gene in our case. However, NUT1, a gene homologous to the major nitrogen regulatory gene [10], is positive for gene rearrangement. It is unknown whether this rearrangement plays any role in the pathogenesis of primay lung bronchogenic squamous cell carcinoma.

## Consent

Written consent was obtained from the patient's parents for publication of this case report. A copy of the written consent is available for review by the Editor-in-Chief of this Journal.

## Competing interests

The authors declare that they have no competing interests.

## Authors' contributions

JFW conceived and drafted the manuscript, BW helped with the photomicrographs, EES and JAJ revised and proof-read the manuscript. DPS reviewed the references, made the final corrections and submitted the manuscript. All authors have read and approved the final manuscript.
